# Promoting patient participation and shortening cancer consultations: a randomised trial

**DOI:** 10.1054/bjoc.2001.2073

**Published:** 2001-09-01

**Authors:** R F Brown, P N Butow, S M Dunn, M H N Tattersall

**Affiliations:** Medical Psychology Unit, Department of Cancer Medicine, University of Sydney; Department of Psychological Medicine, Royal North Shore Hospital, University of Sydney, Camperdown, NSW, Australia

## Abstract

Patient participation in medical consultations has been demonstrated to benefit their subsequent psychological well being. Question asking is one way in which patients can be active. We investigated 2 means of promoting cancer patient question asking. One was the provision of a question prompt sheet to patients prior to their initial consultation with their oncologist. The second was the active endorsement and systematic review of the question prompt sheet by their oncologist. 318 patients with heterogeneous cancers, seeing one of 5 medical and 4 radiation oncologists for the first time, were randomised to either receive or not receive a question prompt sheet. Doctors were randomised to either proactively address or passively respond to the question prompt sheet in the subsequent consultation. Anxiety was assessed prior to the consultation. Consultations were audiotaped and content analysed. Anxiety was assessed again immediately following the consultation. Within the next 10 days patients completed questionnaires assessing information needs, anxiety and satisfaction and were given a structured telephone interview assessing information recall. Patients provided with a question prompt sheet asked more questions about prognosis compared with controls and oncologists gave significantly more prognostic information to these patients. Provision of the question prompt sheet prolonged consultations and increased patient anxiety; however, when oncologists specifically addressed the prompt sheet, anxiety levels were significantly reduced, consultation duration was decreased and recall was significantly improved. A patient question prompt sheet, used proactively by the doctor, is a powerful addition to the oncology consultation. © 2001 Cancer Research Campaign


British Journal of Cancer (2001) 85(9), 1273–1279
© 2001 Cancer Research Campaign
doi: 10.1054/ bjoc.2001.2073, available online at http://www.idealibrary.com on IDEAL

http://www.bjcancer.com

### Promoting patient participation and shortening cancer consultations: a randomised trial

**RF Brown[1], PN Butow[1,2], SM Dunn[1,2] and MHN Tattersall[1,3]**

Medical Psychology Unit[1] and Department of Cancer Medicine[3], University of Sydney, Department of Psychological Medicine[2], Royal North Shore Hospital, University of Sydney, Camperdown NSW, Australia

**Summary** Patient participation in medical consultations has been demonstrated to benefit their subsequent psychological well being. Question asking is one way in which patients can be active. We investigated 2 means of promoting cancer patient question asking. One was the provision of a question prompt sheet to patients prior to their initial consultation with their oncologist. The second was the active endorsement and systematic review of the question prompt sheet by their oncologist. 318 patients with heterogeneous cancers, seeing one of 5 medical and 4 radiation oncologists for the first time, were randomised to either receive or not receive a question prompt sheet. Doctors were randomised to either proactively address or passively respond to the question prompt sheet in the subsequent consultation. Anxiety was assessed prior to the consultation. Consultations were audiotaped and content analysed. Anxiety was assessed again immediately following the consultation. Within the next 10 days patients completed questionnaires assessing information needs, anxiety and satisfaction and were given a structured telephone interview assessing information recall. Patients provided with a question prompt sheet asked more questions about prognosis compared with controls and oncologists gave significantly more prognostic information to these patients. Provision of the question prompt sheet prolonged consultations and increased patient anxiety; however, when oncologists specifically addressed the prompt sheet, anxiety levels were significantly reduced, consultation duration was decreased and recall was significantly improved. A patient question prompt sheet, used proactively by the doctor, is a powerful addition to the oncology consultation. © 2001 Cancer Research Campaign

Cancer patients in Western countries now expect to be fully informed of their diagnosis and involved in decisions about their cancer care (Cassileth et al, 1980; Brody, 1985; Sutherland et al, 1989; Tattersall et al, 1994). When the physician delivers information effectively, patients express higher levels of satisfaction and lower levels of anxiety and distress (Wrigglesmith and Williams, 1975; Stiles et al, 1979; Smith et al, 1981; Blanchard et al, 1990). Recent British research suggests that when general practice patients are unable to voice concerns about issues such as diagnosis, prognosis and treatment side effects during the consultation, they misunderstand information and are less compliant with treatment. It seems that doctors lack confidence in eliciting complex patient agendas and are concerned that addressing these agendas will be overly time consuming (Barry et al, 2000).

Some doctors are concerned that a standard policy of informing patients fully and encouraging them to take an active role in medical decision-making may disadvantage some patients who prefer passivity and cause some patients to become more confused and anxious (Kaplan et al, 1996). Effective and sensitive doctor–patient communication is difficult to achieve. Many studies report that patients are frustrated that they do not obtain the information they require and that doctors are frustrated with patients who do not voice their concerns and requirements for information (Levinson et al, 1993).

One method proposed to encourage patients to better control information flow is increased patient question asking. Patients who actively participate in consultations by asking questions of the doctor are able to change the focus of the consultation and control the duration and the amount of information provided (Kaplan et al, 1996). Street (1991) found that while controlling for other patient factors, the frequency with which patients asked questions was significantly related to the amount of information received about general medical matters and in particular about diagnostic and treatment issues.

Previous attempts to influence patient question asking behaviour have met with limited success (Roter, 1977; Butow et al, 1994; Brown et al, 1999). Roter (1977) evaluated a short coaching session by a psychologist which encouraged general practice patients to ask questions. Although question-asking behaviour was increased, this required considerable resources and one outcome was increased negative interchanges between the doctor and patient. Butow et al (1994) reported the use of a question prompt sheet given to cancer patients before their initial consultation with an oncologist. The question prompt sheet did increase the number of questions about prognosis, although total question asking was unaffected. In a similar study Brown et al (1999) investigated whether combining the previous approaches (coaching and a prompt sheet) would intensify the impact; the question prompt sheet significantly increased the number of questions cancer patients asked generally, and specifically regarding prognosis and tests, but coaching had no additional impact. However, these 3 studies have limitations: (a) the physician was not involved in the intervention process, (b) Roter's study (1977) was conducted in a general practice setting and the generalizability to a cancer setting is questionable and (c) the Butow et al and Brown et al studies (Butow et al, 1994; Brown et al, 1999) were limited to analysis of

*Received 8 February 2001*
*Revised 18 July 2001*
*Accepted 20 July 2001*

*Correspondence to:* RF Brown

This research was funded by the National Health and Medical Research Council of Australia (NH&MRC grant number 950461)

patients from one or two medical oncologists respectively.

The current study investigated the effects of (a) a question prompt sheet provided 15–20 minutes prior to the initial consultation with one of 9 oncologists and (b) active endorsement and systematic review of the question prompt sheet by the physician, on cancer patient question asking, length of the consultation, recall, unmet information needs, anxiety and satisfaction.

### PARTICIPANTS AND METHODS

Patients with heterogeneous cancers, attending an initial consultation with one of 5 medical and 4 radiation oncologists at 2 University teaching hospital outpatient clinics were invited to participate. Exclusion criteria consisted of; (i) age less than 18 years, (ii) non English speaking, (iii) advanced incapacity, (iv) life-threatening illness other than cancer, and (v) non availability for the duration of follow up.

### Procedure

Prior to the consultation patients were informed of the purpose and requirements of the study and written permission was obtained for their participation and to audiotape the consultation. At this time eligible participants were randomly allocated to one of 2 patient groups using a random number table generated by computer.

*Group 1*

Patients were provided with a question prompt sheet (see Figure 1).

*Group 2*

No prompt sheet.

Prior to the commencement of the study the 9 participating doctors were randomly allocated to one of 2 doctor conditions.

*Group 1*

Doctor ‘Passive’; Doctors consulted in their standard manner and were not informed of patient assignment.

*Group 2*

Doctor ‘Proactive’; Doctors were informed when patients had been given a question prompt sheet and they actively addressed the prompt sheet by following a standardised protocol which led them to endorse the importance of asking questions, reassure the patient that they would answer questions to the best of their ability

**How to make the most of your time with the doctor**

Most people who see their doctor for the first time have questions and concerns. Often these get forgotten in the rush of the moment, only to be remembered later. To help you make the most of your time with the doctor we have compiled a list of questions people often ask. We suggest that you tick those you want to ask and then write down any other specific questions you have in the space provided.

You can keep this sheet with you when you see the doctor. You may find that the doctor answers your questions without you even asking, but this sheet can serve as a checklist so that you know that you have covered everything that is important to you.

**Questions people often ask**

1 What kind of cancer do/did I have?
2 Where is the cancer at the moment? Has it spread?
3 What symptoms will the cancer cause?

4 Will I need any more tests?
5 If so, will they hurt?
6 What will they tell us?

7 What treatment will I need?
8 Does the treatment have any side effects? If so, what can be done about them?
9 What should I do or not do while having treatment?
10 How long will it be before I know if the treatment is working?

11 Will my family be affected by my cancer?
12 Will my work be affected?
13 Will my sexual life be affected?

14 What will the outcome be? Will I get better?
15 If we get rid of the cancer, what are the chances of it coming back?

16 Do members of my family have a greater risk of getting cancer?
17 Are there services available to help me cope with this illness?

Write any other questions you have in the space below:

............................................................

............................................................

............................................................

............................................................

**Figure 1** Question prompt sheet

*© 2001 Cancer Research Campaign*

and systematically review each question listed on the question prompt sheet. Doctors received training in the use of the written protocol and after 5 consultations were given feedback about their compliance with suggestions for improvement if necessary. Subsequent analysis of the consultation transcripts revealed that the feedback increased physician compliance with the protocol. Consultations with patients who had not received a question prompt sheet were to be conducted in the doctor's standard manner.

Thus there were 3 groups: (a) patients who did not receive a question prompt sheet and who received standard care (50% of total sample), (b) patients who received a question prompt sheet which was actively endorsed (25% of total sample) and (c) patients who received a question prompt sheet which was not actively endorsed (25% of total sample).

Prior to the consultation, and before randomised patients received the question prompt sheet, all participants completed a short questionnaire measuring anxiety and information and involvement preferences. All consultations were audiotaped to allow analysis of information presented during the consultation. Immediately following the consultation anxiety was re-assessed. All patients were provided with a copy of the tape within one week of the consultation for ethical reasons. Audiotapes were fully transcribed. 7–10 days following the consultation, patients were mailed questionnaires to assess satisfaction with the consultation, anxiety and information needs. Also, within 10 days of the consultation, patients were given a structured telephone interview to assess recall of the information contained within the consultation.

This project received ethical approval from the Central Sydney Area Health Service, Western Sydney Area Health Service and the University of Sydney Ethics Committees.

### Coding

Patient questions (requesting information or guidance) were identified each time they occurred in the transcripts. The content area of the question was coded (history, diagnosis, prognosis, treatment, other medical, psychosocial, social support/counselling/stress management, social exchange, and other/non-specific).

A manual was developed with clear criteria for codes. 2 coders were trained in the use of the manual to code transcripts. Coders re-coded a random 10% of their own consultations and 10% of the other's consultations to determine intra- and inter-rater reliability for the content category within which the question occurred. These proved to be high. The Cohen's Kappa between re-ratings by the same rater on content category was 0.945 and between raters 0.922, respectively. The consultation tapes were timed, as was the length of time the doctor and patient spoke.

### MATERIALS

### Question prompt sheet

The question prompt sheet included text endorsing question asking as an activity useful to the patient and welcomed by their oncologist, followed by a structured list of 17 questions commonly asked by patients of their oncologist. For details of the development of the prompt sheet see Butow et al (1994). Participants were asked to circle those questions they would like to ask and add any additional questions. Patients usually had at least 15 minutes to read and consider the question prompt sheet prior to the consultation (see Figure 1).

### Questionnaires

#### *Anxiety*

Anxiety was measured using the Spielberger State Anxiety Scale (Speilberger, 1983), which is a widely used scale measuring situational anxiety.

#### *Information and involvement preferences*

The amount of information participants required was measured using an adapted form of the Cassileth Information Styles questionnaire (Cassileth et al, 1980). Questions address the amount of detail required (on a 5-point Lickert scale). Patients also indicated their need for more information about 7 specific content areas on a 5-point likert scale. Participants were also asked to indicate their preferred level of involvement in decision making from a range of 5 options (from the doctor only making the decision, to collaborative decision making, to the patient only making the decision) (Degner and Sloan, 1992).

#### *Satisfaction*

Patient satisfaction with the consultation was assessed during the follow-up phase using a 25-item Likert scale adapted from Roter (1977) and Korsch et al (1968). This scale assessed satisfaction with: (i) the amount and quality of information presented; (ii) the communication skills demonstrated by the doctor; and (iii) the level of patient participation in the consultation. The internal reliability of this scale in a sample of 80 patients enrolled in a similar study was high (Cronbach's alpha = 0.91).

#### *Recall*

Recall was measured using a structured telephone interview (Dunn et al, 1993). Recall was assessed by asking questions regarding specific areas which may have been covered during the consultation. Each item recalled by the patient during the telephone interview was recorded and compared with the specific items mentioned by the oncologist during the consultation. The percentage of facts recalled accurately in total, and the number recalled accurately within each category were then calculated.

### Sample size calculation

The primary outcome was total question asking. Calculations are based on our earlier study (Butow et al, 1994) evaluating a question prompt sheet used during consultations with one doctor. Sample size was calculated using the SAM sample calculation package produced by the Australian National Health and Medical Research Council Clinical Trials Centre. The following parameters were used: 80% power, 0.05 level of significance, average question asking of $5.5 \pm 4.5$ rising to 7.5 (0.5 standard deviation) in patients who receive a question prompt sheet. The sample required to detect this difference is 80 per arm. Therefore the required sample size would be 320 patients in total.

### Statistics

Analyses were conducted on an intention-to-treat basis. The total number of questions asked and the number of questions asked within each of the categories of the prompt sheet were calculated. The distributions of all these variables were significantly skewed. Mann–Whitney U tests were applied to detect whether (a) the provision of a prompt sheet or not and (b) the proactive or

© 2001 Cancer Research Campaign passive doctor condition influenced, the total number of questions asked, information needs and satisfaction with the consultation. Kruskall–Wallis one way analysis of variance and Mann–Whitney U tests were used to detect differences in anxiety between groups. Independent sample *t*-tests and one way analyses of variance were used to detect differences in consultation duration and total recall of information as a result of the interventions. Analysis of recall within specific topic areas was conducted using Mann–Whitney U tests due to non-normal distributions.

Demographic and disease variables demonstrating a univariate association below 0.20 with the question-asking outcomes were modelled in multivariate logistic regressions using a backward elimination procedure to determine whether the interventions (question prompt sheet, doctor endorsement) were independent predictors of higher levels of patient outcomes.

### RESULTS

141 female and 177 male cancer patients participated in the study. Participant demographics and disease variables are shown in Table 1. Differences between groups on these variables were non-significant indicating that the randomisation was successful in achieving equal treatment groups.

### Question asking

Patients asked 3493 questions during the 318 consultations (median (md) = 9 per consultation, Inter Quartile Range (IQR) = 0–53). The majority of these questions ($n$ = 1642) related to information about treatment. 122 questions were asked regarding prognosis. The remaining questions were spread across the other content categories (see Table 2). The 318 consultation tapes ranged in duration from 8 minutes to 78 minutes with a mean ($\bar{x}$) duration of 31 minutes (standard deviation (SD) = 13, md = 29.20 minutes).

### Testing for contamination effects

Our first concern was to test whether doctors were able to maintain their standard practice when the randomisation dictated. If so, active doctors should maintain their standard practice with those patients who did not receive the question prompt sheet, while passive doctors should behave in their standard manner with all patients, whether they received a prompt sheet or not.

**Table 1** Demographic and disease characteristics of sample ($n$ = 318)

| | | |
|---|---|---|
| Age | | |
| | Mean | $\bar{x}$ = 56.12 years |
| | Range | 18–83 years |
| Gender | | |
| | Female | 141 (44.3%) |
| | Male | 177 (55.7%) |
| Education level | | |
| | Completed < ten years | 86 (27%) |
| | Completed High School | 132 (41.5%) |
| | Tertiary – Non University | 30 (9.4%) |
| | University | 64 (20.1%) |
| | Unknown | 6 (1.9%) |
| Occupation | | |
| | Professionals | 123 (38.7%) |
| | Trades People | 40 (12.6%) |
| | Clerks and Sales | 72 (22.6%) |
| | Labourers | 50 (15.7%) |
| | Home duties/Students | 20 (6.3%) |
| | Unknown | 13 (4.1%) |
| Marital status | | |
| | Single | 42 (13.2%) |
| | Married | 199 (62.6%) |
| | Divorced/Separated | 41 (12.9%) |
| | Common Law | 8 (2.5%) |
| | Widowed | 26 (8.2%) |
| | Unknown | 2 (0.6%) |
| Type of cancer | | |
| | Breast | 62 (19.5%) |
| | Gastrointestinal | 55 (17.3%) |
| | Lymphoproliferative | 42 (13.2%) |
| | Genitourinary | 67 (21.0%) |
| | Skin | 45 (14.2%) |
| | Other | 47 (14.8%) |
| Disease status | | |
| | Loco-Regional | 179 (56.9%) |
| | Metastasis | 114 (35.8%) |
| | Unknown | 23 (7.2%) |
| Estimated prognosis | | |
| | < 1 year | 95 (29.8%) |
| | 1–5 years | 156 (49.3%) |
| | Normal life expectancy | 44 (13.9%) |
| | Unknown | 22 (6.9%) |

Prior to the commencement of the study, 5 baseline consultations of each doctor were audiotaped. These consultations were

**Table 2** Frequencies of questions in each category by group

| | Control $n$ = 158 | | Passive Doctor +QPS $n$ = 79 | | Proactive Doctor +QPS $n$ = 81 | | Total | |
|---|---|---|---|---|---|---|---|---|
| | No of questions | % of group | No of Questions | % of group | No of Questions | % of group | Sum | % of total |
| **Category** | | | | | | | | |
| History | 155 | 8.9 | 52 | 6.1 | 60 | 6.8 | 267 | 7.6 |
| Diagnosis | 220 | 12.6 | 105 | 12.3 | 97 | 10.9 | 422 | 12.1 |
| Prognosis | 124 | 7.1 | 91 | 10.5 | 107 | 12.0 | 322 | 9.2 |
| Treatment | 867 | 49.6 | 395 | 46.1 | 380 | 42.8 | 1642 | 47.0 |
| Other medical | 151 | 8.7 | 79 | 9.2 | 127 | 14.3 | 357 | 10.2 |
| Psychosocial issues | 119 | 6.8 | 80 | 9.3 | 62 | 7.0 | 261 | 7.5 |
| SS/C/SM[a] | 2 | 0.1 | 3 | 0.4 | 2 | 0.2 | 7 | 0.2 |
| Social exchange | 7 | 0.4 | 0 | 0 | 2 | 0.2 | 9 | 0.3 |
| Other/non-specific | 102 | 5.8 | 52 | 6.1 | 52 | 5.8 | 206 | 5.9 |
| Total | 1747 | 100 | 857 | 100 | 889 | 100 | 3493 | 100 |

[a]Social Support/Counselling/Stress Management.

 *© 2001 Cancer Research Campaign*

transcribed in full and patient questions were identified and counted. Patient question asking was compared for active doctors only, between baseline consultations and those in which patients did not receive a question-prompt sheet, using a Man–Whitney U test. No significant differences were found, suggesting that active doctors were able to maintain their standard practice as directed. Similarly, we compared question asking for passive doctors only, for consultations in which patients received or did not receive a question prompt sheet. No significant differences were found, suggesting that passive doctors also were able to maintain their standard practice.

### Impact of the prompt sheet and doctor intervention on question asking

In patients who had a prompt sheet, no significant differences were detected in the total number of questions asked between those who saw a passive or proactive doctor. The same was true of the number of questions asked in each of the content categories. Thus these 2 groups were collapsed to allow an exploration of differences in question asking between patients with a prompt sheet or not. No significant differences were found in the total number of questions asked; however, patients with a prompt sheet (md = 0, IQR = 0–2) asked significantly more questions regarding prognosis than those without (md = 0, IQR = 0–1) ($z = -2.07$, $P = 0.039$). The prognosis category included any questions about the likely course and outcome of the disease, risk of relapse and chances of cure.

Additionally, the total number of items of prognostic information given by the doctors was significantly more for patients with a prompt sheet (md = 5, IQR = 3–8) than those without (md = 4, IQR = 2–7) ($z = -2.91$, $P = 0.004$).

We then explored the hypothesis that the question prompt sheet had a greater impact on question asking about prognosis if the doctor did not often talk about prognosis in standard consultations. Doctors were coded into high and low providers of prognostic information (providing above or below a median of 4 items of prognostic information in standard consultations where the question prompt sheet was not used). An interaction term was calculated between this variable and the question prompt sheet variable. However the interaction term proved to be non-significant in logistic regression, indicating that the question prompt sheet did not have a greater impact when the doctor did not often talk about prognosis.

### *Multivariate analysis of factors influencing question asking about prognosis*

As prognosis was the only category in which question asking was influenced by the provision of a question prompt sheet, multivariate analyses were conducted only for this category. Occupation, education, age and stage of disease were associated with question asking about prognosis below the 0.20 level of significance and were therefore included in the multivariate model. Question asking about prognosis was recoded into a dichotomous dependent variable (0 vs 1 or more questions).

Following the logistic regression only the prompt sheet variable remained in the model. Patients with a prompt sheet were more likely to ask a question about prognosis (Odds ratio (OR) = 1.60, 95% CI = 0.98–2.60) than control patients; however, this result can only be reported as a strong trend to significance ($P = 0.058$).

### Impact of the prompt sheet and doctor interventions on consultation length

The consultations of radiation oncologists, in this study, tended to be shorter (mean = 23.27 min, SD = 9.2 min) than the consultations of medical oncologists (mean = 38.9 min, SD = 11.1 min). The length of consultations ranged between 8 to 78 minutes. The presence of 5 outliers in the medical oncology consultations accounted for much of the skew in the distribution. Significant differences were detected in consultation length as a result of the interventions ($F_{2, 302} = 3.89$, $P = 0.021$). Tukey's post-hoc comparison testing indicated that consultations in which the doctor was proactive and the patient had a prompt sheet were significantly shorter ($\bar{x}$ = 28.50 minutes, SD = 9.87) than those in which the patient had a prompt sheet alone ($\bar{x}$ = 34.36 minutes, SD = 14.93) and the control group ($\bar{x}$ = 32.09 minutes, SD = 13.13).

### Information needs

Frequency of response was explored for each of the 7 items comprising the Information Needs Scale. Relatively high unmet needs were reported, especially on some items. For example, 30% of patients wanted more information about prognosis after the consultation. A total score was computed for this scale by summing those items where information was still desired after the consultation. Neither the prompt sheet nor doctor endorsement were associated with unmet need for information.

### Recall

Patients who had a prompt sheet and whose doctor was proactive ($\bar{x}$ = 52%, SD = 18) recalled significantly more information in total than those patients with a prompt sheet alone ($\bar{x}$ = 44%, SD = 21) ($t_{126} = -2.118$, $P = 0.036$). The no prompt sheet group was not significantly different from the other groups. When specific content categories were explored, patients with a prompt sheet whose doctor was proactive recalled significantly more information regarding treatment issues ($z = 6.606$, $P = 0.010$) and side effects ($z = -2.608$, $P = 0.009$) than those with a prompt sheet alone.

### *Multivariate analysis of factors influencing total recall*

Patient age, gender and whether the patient had listened to the consultation audiotape prior to the recall interview were associated with total recall below the 0.20 level of significance. Interaction terms were calculated between the patient age and gender variables and the endorsement of the prompt sheet variable. These variables were included in the multivariate linear regression model. Only the interaction between gender and endorsement of the prompt sheet variable remained in the model ($F_{1, 186} = 6.056$, $P = 0.015$), indicating that males whose doctor endorsed the prompt sheet recalled more information while this was not true for females.

### Anxiety

Anxiety levels were equal between the patient groups prior to the consultation. Immediately following the consultation patients with a prompt sheet alone (md = 37, IQR = 28–46) were significantly more anxious than either patients with a prompt sheet and a proactive doctor (md = 31, IQR = 22–40) or patients in the control group (md = 32, IQR = 25–43) ($\chi^2_2 = 8.607$, $P = 0.041$). The latter 2 groups

© 2001 Cancer Research Campaign were not significantly different. There were no differences in anxiety levels between the groups at the one week post-consultation measurement.

### Satisfaction

There were no differences in satisfaction as a result of the prompt sheet or doctor endorsement.

### DISCUSSION

Patients provided with a question prompt sheet compared to controls were more likely to ask a question about prognosis although this did not quite reach significance after controlling for sociodemographic and disease variables. Oncologists also gave significantly more prognostic information to patients with a prompt sheet. These effects were apparent even in consultations with doctors who usually discussed prognosis. This simple intervention had no similar impact on patient question asking about other items discussed in initial consultations with oncologists.

Provision of a question prompt sheet to cancer patients before an initial consultation has been found in 2 separate studies to promote question asking about prognosis (Butow et al, 1994; Brown et al, 1999). The significant impact in this study appeared to be in increasing the percentage of patients who asked at least one question about prognosis (with 48% of patients with a prompt sheet asking at least one question about prognosis, compared to 39% of those without). Arguably, one question is all that is required to produce the information needed.

It is unclear whether the change is due to the prompt 'legitimising' question asking in this sensitive area, or whether it reflects oncologist reluctance to spontaneously raise this matter at an initial consultation even though cancer management recommendations are frequently made at this time. However, the fact that no overall increase in question asking was observed in patients receiving a prompt sheet suggests that the prompt sheet 'empowers' patients to ask questions which focus discussion on a topic which is not usually adequately discussed by oncologists in their initial consultations. In another study analysing taped consultations, we have documented that only 43% of patients with metastatic disease received information about their prognosis in the initial consultation (Gattellari et al, 1999), while the number of patients receiving information about life expectancy with and without treatment was even smaller (14%). In another Australian study only 43% of 172 breast and melanoma patients of all stages, interviewed 6–12 months after their diagnosis, reported that they had been told their prognosis (Butow et al, 1996).

Reticence to provide information about prognosis to patients is often based on a concern that the information could be contrary to patients' wishes or best interests, and further, that once raised, the doctor will be unable to provide appropriate support. Reassuringly, in this study while patients provided with a prompt sheet were significantly more anxious immediately following the consultation than those without, anxiety levels were significantly lower in the former group when the oncologist specifically addressed questions in the prompt sheet during the consultation. Together these results suggest that the prompt sheet promotes patient confidence to ask this difficult question, and provided the oncologist specifically addresses this and other issues raised in the question prompt sheet, patient anxiety is ameliorated.

A further concern expressed by doctors about discussing prognosis, is that in subsequently addressing the issues raised, the consultation will be excessively lengthened. In busy clinics, even 10 minutes added per consultation can cause havoc. Again this study provides reassurance on this point. The broad range of consultation lengths can be explained by the presence of several outliers in the medical oncology consultations. Long consultations were not typical of the sample. Provision of the question prompt sheet prolonged consultations, but if the oncologist addressed the sheet during the consultation, the average consultation length was significantly reduced, and to a shorter average duration than consultations without a question prompt sheet. This finding suggests that inviting patients to prepare for their consultation by proposing possible questions and then formally addressing them, can assist in organising the consultation more efficiently, and avoiding circuitous discussion while the patient tries to clarify his or her concerns, even if difficult issues are raised.

Finally, patients given a question prompt sheet which the oncologist addressed also recalled significantly more information about treatment issues and side effects than patients with a prompt sheet alone. This finding suggests that oncologist endorsement of question asking and their attention to issues raised in the prompt sheet reinforce treatment information which otherwise may not be recalled. Men recalled more information than women and appeared to benefit more from the doctor endorsing the question prompt sheet than did women. This result is hard to explain and deserves further exploration.

Despite being an advance on previous studies which have involved only one or two doctors, this study still included only 9 oncologists and the patients in their practice. The doctors involved came from 2 large urban centres; therefore the majority of patients were urban although a minority were seen at a rural outpatient clinic. Furthermore, a large percentage of the respondents had completed tertiary education (32%) and had a professional occupation (39%). Education and occupation proved in univariate analyses to be associated with question asking about prognosis. Therefore, the generalisability of the findings to the larger oncology community is questionable. Replication in other settings is recommended.

In summary, a question prompt sheet, if addressed in the consultation, shortens consultations, enhances recall and reduces patient anxiety. It is plausible that the provision of a question prompt sheet may have broad application in general medical practice. This simple intervention seems to have a lot going for it!

### ACKNOWLEDGMENTS

We gratefully acknowledge the participation of the patients and the contribution of the oncologists involved in this research at Royal Prince Alfred and Westmead Hospitals; Dr Michael Barton, Dr Michael Boyer, Professor Alan Coates, Dr Michael Findlay, Dr Craig McCleod, Dr Susan Pendlebury, Dr Anne Sullivan and Dr Sandra Turner. We also gratefully acknowledge the contribution to coding and data collection of Ms Elizabeth Dent and Ms Melina Gattelari.

### REFERENCES

Barry CA, Bradley CP, Britten N, Stevenson FA and Barber N (2000) Patients unvoiced agendas in general practice consultations: qualitative study. *BMJ* **320**: 1246–1250

Blanchard CG, Labreque BA, Ruckdeschel JC and Blanchard EB (1990) Physician behaviours, patient perceptions and patient characteristics as predictors of satisfaction of hospitalized cancer patients. *Cancer* **65**: 186–192

*© 2001 Cancer Research Campaign*

Brody H (1985) Autonomy revisited: progress in medical ethics. *J Roy Soc Med* **78**: 380–387

Brown RF, Butow PN, Boyer M and Tattersall MHN (1999) Promoting patient participation in the cancer consultation: evaluation of a prompt sheet and coaching in question asking. *Br J Cancer* **80**(1/2): 242–248

Butow PN, Dunn SM, Tattersall MHN and Jones QJ (1994) Patient participation in the cancer consultation: Evaluation of a question sheet. *Annals of Oncology* **5**: 199–204

Butow PN, Kazemi J, Beeney LJ, Griffin A-M, Dunn SM and Tattersall MHN (1996) When the diagnosis is cancer: Patient communication experiences and preferences. *Cancer* **77**(12): 2630–2637

Cassileth BR, Zupkis RV, Sutton-Smith K and March V (1980) Information and participation preferences among cancer patients. *Annals of Internal Medicine* **92**: 832–836

Dunn SM, Butow PN, Tattersall MHN, Jones QJ, Sheldon J, Taylor J and Sumich MD (1993) General information tapes inhibit recall of the cancer consultation. *Journal of Clinical Oncology* **11**(11): 2279–2285

Gattellari M, Butow PN and Tattersall MHN (1999) Informed consent: what did the doctor say? *Lancet* **353**: 1713

Kaplan SH, Greenfield S, Gandek B, Rogers WH and Ware JE (1996) Characteristics of physicians with participatory decision making styles. *Annals of Internal Medicine* **124**: 497–504

Korsch BM, Gozzi EK and Francis V (1968) Gaps in Doctor – Patient communication: Doctor patient interaction and patient satisfaction. *Pediatrics* **42**(5): 855–870

Levinson W, Stiles WB, Inui TS and Engle R (1993) Physician frustration in communicating with patients. *Medical Care* **31**: 285–295

Roter D (1977) Patient participation in the patient-provider interaction: the effects of patient question asking on the quality of interaction satisfaction and compliance. *Health Education Monographs* **5**: 281–315

Smith CK, Polis E and Hadac RR (1981) Characteristics of the initial medical interview associated with patient satisfaction and understanding. *The Journal of Family Practice* **12**(2): 283–288

Speilberger CD (1983) Manual for the state trait anxiety inventory (Form Y). *Consulting Psychologists Press*. Palo Alto. CA

Stiles WD, Putnam SM, Wolf MH and Sherman JA (1979) Interaction exchange structure and patient satisfaction with medical interviews. *Medical Care* **17**(6): 667–679

Street RL (1991) Information giving in medical consultations: The influence of patient communicative styles and personal characteristics. *Social Science and Medicine* **32**(5): 541–548

Sutherland HJ, Llewellyn-Thomas HA, Lockwood GA and Trichler DL (1989) Cancer patients: their desire for information and participation in treatment decisions. *J Roy Soc Med* **82**: 260–263

Tattersall MHN, Butow PN, Griffin AM and Dunn SM (1994) The take home message: patients prefer consultation audiotapes to summary letters. *Journal of Clinical Oncology* **12**(6): 1305–1311

Wrigglesmith J and Williams J (1975) The construction of an objective test to measure patient satisfaction. *Int J Nursing Studies* **12**: 123–132

*© 2001 Cancer Research Campaign*
